# FUNYBASE: a FUNgal phYlogenomic dataBASE

**DOI:** 10.1186/1471-2105-9-456

**Published:** 2008-10-27

**Authors:** Sylvain Marthey, Gabriela Aguileta, François Rodolphe, Annie Gendrault, Tatiana Giraud, Elisabeth Fournier, Manuela Lopez-Villavicencio, Angélique Gautier, Marc-Henri Lebrun, Hélène Chiapello

**Affiliations:** 1 UR MIG, INRA, Bâtiment 233 Domaine de Vilvert 78350, Cedex, France; 2 UMR ESE, Université Paris-Sud, CNRS, AgroParisTech, Bâtiment 360, Université Paris-Sud, 91405 Orsay, Cedex France; CNRS 91405 Orsay, France; 3 UMR BGPI, INRA, CIRAD, AgroSup, TA A 54/K, Campus International de Baillarguet, 34398, Montpellier, cedex 5, France; 4 MNHN, Département Systématique et Evolution, 12 rue Buffon CP 39, 75005 Paris, France; 5 UMR BIOGER, INRA, AgroParisTech, Centre INRA de Versailles, Route de Saint Cyr 78026 ,Versailles, France; 6 UMR MAP Université Lyon-1, CNRS, INSA, BAYER CS, 14, rue Pierre Baizet 69009 Lyon, France

## Abstract

**Background:**

The increasing availability of fungal genome sequences provides large numbers of proteins for evolutionary and phylogenetic analyses. However the heterogeneity of data, including the quality of genome annotation and the difficulty of retrieving true orthologs, makes such investigations challenging. The aim of this study was to provide a reliable and integrated resource of orthologous gene families to perform comparative and phylogenetic analyses in fungi.

**Description:**

FUNYBASE is a database dedicated to the analysis of fungal single-copy genes extracted from available fungal genomes sequences, their classification into reliable clusters of orthologs, and the assessment of their informative value for phylogenetic reconstruction based on amino acid sequences. The current release of FUNYBASE contains two types of protein data: (i) a complete set of protein sequences extracted from 30 public fungal genomes and classified into clusters of orthologs using a robust automated procedure, and (ii) a subset of 246 reliable ortholog clusters present as single copy genes in 21 fungal genomes. For each of these 246 ortholog clusters, phylogenetic trees were reconstructed based on their amino acid sequences. To assess the informative value of each ortholog cluster, each was compared to a reference species tree constructed using a concatenation of roughly half of the 246 sequences that are best approximated by the WAG evolutionary model. The orthologs were classified according to a topological score, which measures their ability to recover the same topology as the reference species tree. The full results of these analyses are available on-line with a user-friendly interface that allows for searches to be performed by species name, the ortholog cluster, various keywords, or using the BLAST algorithm. Examples of fruitful utilization of FUNYBASE for investigation of fungal phylogenetics are also presented.

**Conclusion:**

FUNYBASE constitutes a novel and useful resource for two types of analyses: (i) comparative studies can be greatly facilitated by reliable clusters of orthologs across sets of user-defined fungal genomes, and (ii) phylogenetic reconstruction can be improved by identifying genes with the highest informative value at the desired taxonomic level.

## Background

Since the historical genome sequencing of the yeast *Saccharomyces cerevisiae *in 1996 [1], a large increase in the number of available fungal genomes has occurred, especially during the last five years. This is partly due to the small size of fungal genomes and the role of consortia such as the Fungal Genome Initiative at the Broad Institute, the Eukaryotic Genomics Initiative at the JGI, the TIGR and Genoscope sequencing projects. Consequently, more than 60 fungal genomes are now publicly accessible [2,3], making this group one of the best-represented eukaryotic phyla with regard to available genomic data.

This rapid increase in fungal genome sequences has identified a very large number of genes useful for comparative analyses. Such studies generally require the non-trivial task of first assigning genes to protein families according to a criterion reflecting the observed sequence diversity. The most common metrics for this classification are either the percent identity deduced from pair-wise amino acid sequence alignments or the BLAST e-value. The most common methods to produce sets of orthologous proteins are generalized simple link classifications, generalized bi-directional best-hit, or more sophisticated algorithms like the Markov Cluster Algorithm [4,5]. However, the choice of a clustering algorithm may greatly impact subsequent analyses [6]. This step can be influenced by biases like the quality of genome annotation (i.e. the accuracy of gene prediction) and the presence of multi-domain proteins which can possibly generate artificial clusters of homologous sequences.

A growing number of online resources are providing access to genome sequences, such as the Fungal Genome Intiative (FGI) at the Broad Institute, the Eukaryotic Genomics Database at the JGI, the TIGR fungal database, the NCBI Entrez database, or the MIPS fungal database, to name a few. Several databases have been recently developed to specifically facilitate comparative analysis in fungi. Most of these resources are dedicated to a particular taxonomic group, such as hemi-ascomycetes [7], including yeast (Saccharomyces Genome Database) and *Candida *[8]. A few are generalist resources integrating all public fungal genomes, including the e-Fungi repository [9]. This latter database includes virtually all fungal genomes and ESTs regardless of their sequence quality and annotation reliability. Finally, the AFTOL (Assembling the Fungal Tree Of Life, ) database was recently developed to provide easy access to the fungal tree life database via the WASABI (Web Accessible Sequence Analysis for Biological Inference) system [10]. One of the goals of AFTOL is to make sequence data, alignments, and other types of data rapidly and broadly available to the scientific community.

The increasing number of available fungal genome sequences is also very valuable to efforts in robust phylogenetic reconstruction. Indeed, the reliability of the species trees to depict actual evolutionary relationships increases when using multiple independent loci, while phylogenies based on one or a few genes can be misleading [11]. Several recent studies have used complete genome sequences to build robust fungal phylogenies [3,11-14]. However, if we are to reconstruct phylogenetic relationships among fungal species whose complete genomes are not sequenced, only a limited number of DNA fragments can be practically sequenced. It is therefore useful to many studies if individual genes can be identified that would best reflect the phylogenetic tree based upon the proper alignment of the genome as a whole. Additionally, if we aim to estimate phylogenies among closely related species, or among isolates from a single species, it is useful to know which genes have a high rate of divergence or which ones have an optimal evolutionary rate for resolving relationships at particular taxonomic scales [15].

Here, we present a novel online database and analysis gateway, FUNYBASE, useful for comparative genomics and phylogenetic analyses of Fungi, which does not focus on any particular group or phylum of the kingdom. We have used a robust approach based on BLAST comparisons and followed by a Markov Cluster Algorithm classification to determine reliable clusters of single-copy orthologous genes in fungi that are necessary for comparative and evolutionary genomics. Furthermore, the database provides a measure of the informative value of each gene for phylogenetic reconstruction, i.e. the ability of each gene to yield a phylogenetic tree reflecting larger-scale genome relatedness [11]. Unlike other fungal databases, we also provide data from phylogenetic analyses, such as alignment statistics, estimated tree, and evolutionary model fitting for each ortholog cluster.

## Construction and content

### Data sources

Our initial dataset contained 30 fungal genomes (ascomycetes, basidiomycetes, and zygomycetes) (see Table 1). Genome sources were: NCBI, JGI, BROAD, and Washington University. This dataset corresponds to 275,948 predicted proteins.

**Table 1:** Fungal genome sources

**Species**	**Source**	**Nb proteins**	**Release or Date**	**Online database**
*Ashbya gossypii*	AGD	4726	2.1	
*Aspergillus fumigatus*	NCBI	9923	06/30/2006	
*Aspergillus nidulans*	BROAD	10701	1	
*Aspergillus oryzae*	NITE	12074	07/13/2006	
*Botrytis cinerea*	BROAD	16448	1	
*Candida glabrata*	NCBI	5181	07/05/2006	
*Candida lusitaniae*	BROAD	5941	1	
*Chaetomium globosum*	BROAD	11124	1	
*Coccidioides immitis*	BROAD	10457	2	
*Cryptococcus neoformans*	NCBI	6475	07/30/2006	
*Debaryomyces hansenii*	NCBI	6317	07/30/2006	
*Eremothecium gossypii*	NCBI	4718	07/30/2006	
*Fusarium graminearum*	BROAD	11640	1	
*Kluyveromyces lactis*	NCBI	5331	07/30/2006	
*Magnaporthe grisea*	BROAD	12841	5	
*Neurospora crassa*	BROAD	10620	7	
*Phanerochaete chrysosporium*	JGI	10048	2.1	
*Rhizopus oryzae*	BROAD	17467	3	
*Saccharomyces bayanus*	MIT	9424	07/13/2006	
*Saccharomyces castellii*	WashU	4677	07/13/2006	
*Saccharomyces cerevisiae*	NCBI	5869	07/30/2006	
*Saccharomyces kluyveri*	WashU	2968	07/13/2006	
*Saccharomyces kudriavzevi*	WashU	3768	07/13/2006	
*Saccharomyces mikatae*	MIT	9057	07/13/2006	
*Saccharomyces paradoxus*	MIT	8955	07/30/2006	
*Schizosaccharomyces pombe*	NCBI	5045	07/30/2006	
*Sclerotinia sclerotiorum*	BROAD	14522	1	
*Stagonospora nodorum*	BROAD	16597	1	
*Trchoderma reesei*	JGI	9997	1.2	
*Ustilago maydis*	BROAD	6522	1	
*Yarrowia lipolytica*	NCBI	6520	07/30/2006	

### Construction of protein families

A BLASTP search of each predicted protein sequences against the entire assembled protein sequences database was performed using the NCBI BLAST2 software [16]. Alignments were considered non-spurious after HSP-tiling if they met three criteria: (i) coverage of at least 70% of the query sequence, (ii) identity of at least 30%, and (iii) E-value cutoff of 6e-6. The BLAST results were analyzed with the program Tribe-MCL obtained from [17]. The program Tribe-MCL uses Markov Clustering (MCL) by creating a similarity matrix from BLAST e-values and then clusters proteins into related groups. The main parameter that influences the size of a cluster in Tribe-MCL is the inflation value, which can be adjusted from 1.1 (fewer clusters are formed but with more proteins in each) to 5.0 (more but smaller clusters are formed and proteins with high similarity remain clustered together). In order to obtain robust ortholog clusters corresponding to single copy genes present in all fungal genomes, we used the stringent inflation value of I = 4 and filtered clusters that contain exactly one protein per fungal genome (hereafter referred to as single-copy clusters).

### Database design

FUNYBASE is implemented on the relational database system PostgreSQL (version 8.2.4). Custom-made parsers have been developed to integrate genomes, annotations, BLAST results and MCL clusters in the database. All parsers were developed in Perl using standard modules, such as BioPerl, DBI and POD documentation (available on request). The Web interface is designed using the standard Perl modules DBI and CGI.

### Content

**Figure 1:**
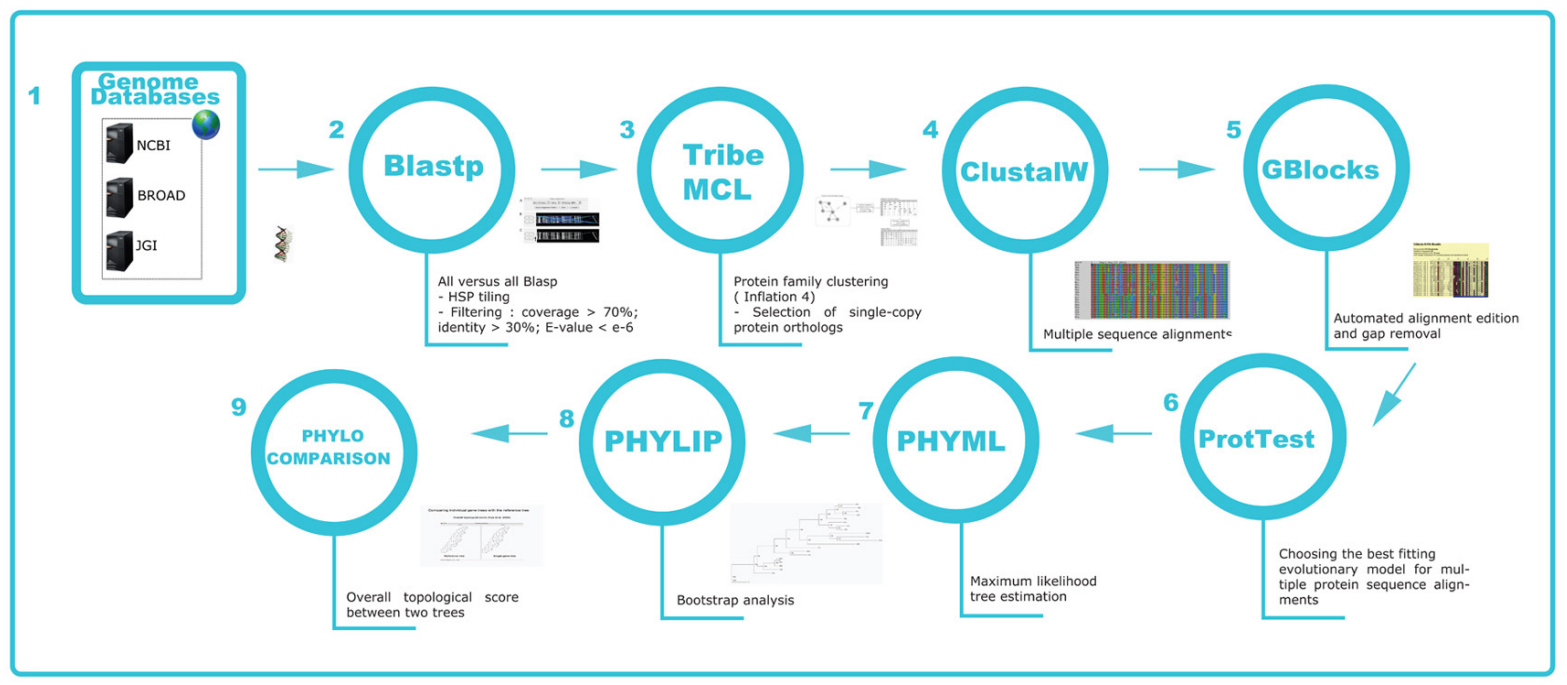
**FUNYBASE Pipeline.** Scheme showing the main steps in the construction of the ortholog clusters and their subsequent phylogenetic analysis (for more details see [10]).

FUNYBASE includes two sets of data:

- the complete protein clusters dataset, including orthologs and paralogs, built from the 30 available fungal genomes,

- the subset of 246 families of single-copy orthologs obtained from 21 genomes with which further phylogenetic analyses were performed (Fig. 1) [11]. This subset of 21 genomes was chosen as a set of fungal genome sequences with reliable gene prediction (see Ref. [11] for more details). For each of these 246 ortholog clusters, FUNYBASE provides the amino-acid substitution model that best fits the data, the available annotation for the family, the mean identity percentage of the sequences in the family, the number of variable sites, the aligned proteins, the corresponding phylogenetic tree, and its similarity with the tree resulting from the concatenated dataset (i.e., its topological score, and index going from 0 to 100, see Ref. [11] for more details).

### Web interface (Fig. 2 and 3)

**Figure 2:**
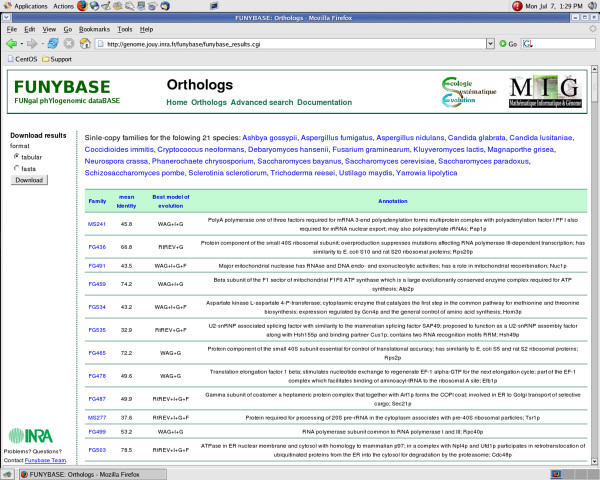
**a) FUNYBASE Orthologs Page.** Entries include "Ortholog Family Name", "Mean Identity", "Best Model of evolution" and "Annotation".

**Figure 3:**
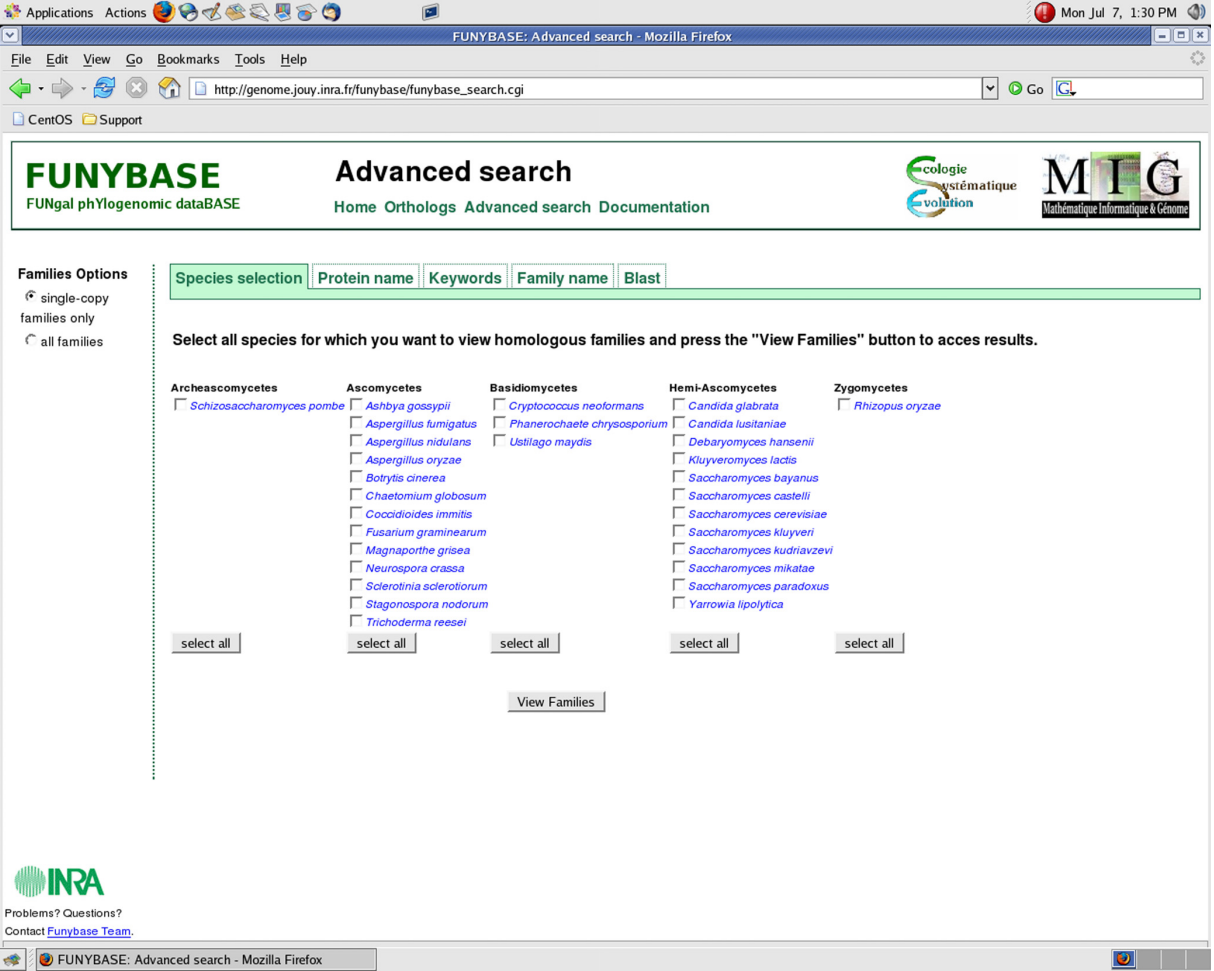
**b) FUNYBASE Advanced Search Page.** One can select the orthologs from a specific species, or group of species. Options for viewing include "Single-Copy Families Only" or "All Families".

The database can be accessed through two main Web pages:

- the "Orthologs" page provides detailed information on the 246 families of single copy orthologs obtained from the 21 genomes with reliable gene annotations (Fig. 2),

- the "Advanced Search" page provides addition methods (detailed below) for accessing protein families defined from the 31 public complete fungal genomes (Fig. 3).

#### The "Orthologs" page

The "Orthologs" page contains detailed information on the 246 families of single-copy orthologs described previously [11]. These families contain orthologs common to the subset of 21 genomes. By clicking on the "Orthologs" link in the main banner, a table can be obtained which describes the 246 single-copy families. The families can be sorted out using different criteria by clicking on the column titles of the table. For each single-copy family, the following information can be obtained:

(1) the family name,

(2) the mean identity percentage within the family (based on the ClustalW aligment),

(3) the best model of evolution: a probabilistic model that describes the different probabilities of change from one amino-acid, or codon, to another. The different parameters of the model aim at integrating the factors involved in the substitution process. In order to choose the best model for a given dataset (multiple sequence alignment), we used the program ProtTest that ranks the models according to the AIC or BIC criteria [18].

(4) the protein cluster annotation.

By clicking on a family name, it is possible to obtain detailed information on one cluster, including:

- Topological Scores [19]: this index is estimated by pairing all the branches that are shared between the gene tree and species tree based on the concatenated dataset and building a 1-to-1 optimum map that takes into account the differences in terms of topology and branch lengths (see Ref. [11] for more details).

- Average Rates: the mean posterior estimation of the number of substitutions per site, as obtained by maximum likelihood using the PAML software [20].

- List of proteins from a family and their annotations.

- ClustalW aligments, which can be downloaded (Phylip format).

- Phylogenetic trees, which can be download (in Cladogram or Newick format).

#### The Advanced Search page

The five ways of accessing data on ortholog clusters in the "Advanced search" mode are:

(1) 'Species selection': This section allows selecting either a single family of orthologous genes or all families for a given group of species.

(2) 'Protein name': This section makes it possible to find a family containing a given protein identified by a protein ID.

(3) 'Keywords': This interface allows the user to find all the families that contain at least one protein whose annotation matches the queried keyword.

(4) 'Family name': This section allows the user accessing a family (or families) using its name.

(5) 'BLAST': This section allows performing a BLAST (either BLASTP or BLASTX) comparison between a query sequence and the complete FUNYBASE comprising all the proteins deduced from the 31 public fungal genome sequences. The produced BLAST results contain links with an access to the protein family corresponding to the hit.

## Utility and Discussion

### Reliability of the ortholog clusters

To identify clusters of orthologous genes, we used MCL clustering methods to recover the maximum number of orthologous gene clusters with sufficiently stringent parameters to avoid families containing hidden paralogs. This approach differs significantly from those used to develop other databases and interactive web tools. The trade-off involved in recovering reliable ortholog clusters is best handled with MCL because this method can be finely tuned with respect to the dataset [21,22]. We chose a value of the inflation parameter that had been shown to produce an optimal number of clusters containing orthologous single-copy genes [4,7,13]. According to Robbertse et al. [13], the number of orthologous gene clusters found in available fungal genomes reaches a constant value when increasing the inflation parameter over three, suggesting that the value of four we chose experimentally is appropriate. Other studies used rather *ad hoc *methods to obtain clusters of orthologous genes, either identifying families with related genes present as a single copy in each genome analyzed [12] or inferring orthology based on the KOG database [17].

We consider that these *ad hoc *methods are not efficient in detecting clusters of reliable single-copy orthologous genes. For instance, definitions of orthology can be too liberal if all that they require is that a gene be present only once in all compared genomes, as hidden paralogy can pose a serious problem. On the other hand, some methods can be too conservative if they are based on similarity searches using more general databases, such as KOG, which currently includes only two fungal genomes (*S. cerevisiae *and *S. pombe*) and requires similarity with more distantly related eukaryotes, resulting in the systematic exclusion of the orthologs shared exclusively by fungi. Also, many artefacts can be produced if methods fail to take into account the modular structure of proteins, which may result in the false-positive clustering of orthologs, especially in the case of multi-domain proteins.

Clustering methods come in two general flavors, as they are either based on similarity searches (e.g., BBH, KOGs, INPARANOID [23], RSD [24], Tribe-MCL, Ortho-MCL), or are tree-based (i.e., they take into account the phylogenetic relationships between orthologs and paralogs). If a reliable species phylogeny is available, tree-based methods may be more accurate in the resolution of homology relationships because phylogenies naturally portray information on lineage-specific duplications and losses. The most significant drawback of tree-based methods is the intensive computation time required and the expert curation needed to evaluate the correct phylogenetic inference of gene families. A recently proposed method may alleviate some of these burdens by using a mixed approach, including similarity searches and tree-based methods at different stages of the analysis (e.g., SYNERGY [25]). However, tree-based methods rely on the assumption that there is a robust species tree available. Since many studies do not have any *a priori *species tree, it is often essential to take advantage of the best clustering method that makes no assumptions about a pre-specified phylogeny (i.e., MCL clustering methods).

### Usefulness for genomics

FUNYBASE provides an important resource for fungal comparative genomics, as it allows the retrieval of clusters of orthologs shared among 21 species, representing the major fungal taxonomic groups across a large phylogenetic scale. This information can serve multiple purposes, including:

#### Gene comparison

gene sequences, general descriptions, statistics and alignments of the 246 clusters of orthologous genes are available for direct comparison. The molecular evolution of a given gene, or set of genes, can be obtained at any taxonomic level. Moreover, it is possible to highlight different levels of gene conservation and/or divergence among fungal lineages in order to assess lineage-specific or gene-specific evolutionary patterns.

#### Tree comparison

the phylogenetic gene trees corresponding to the 246 clusters of orthologous genes are available and can be directly employed to test different evolutionary hypotheses. Comparisons of the tree topologies can be used for different evolutionary studies, such as finding evidence for incomplete lineage sorting, horizontal gene transfers, or accelerated evolutionary rates in some gene families.

#### Gene searching

FUNYBASE allows BLAST searches against the set of protein sequences corresponding to the 246 clusters of orthologous genes. Alignments of protein sequences from one cluster can be used to construct Hidden Markov Model (HMM) profiles for HMM-based searches of the corresponding orthologous genes in novel genome sequences.

#### Gene function prediction

it is possible to use proteins from novel genomes as queries to find matching annotated sequences in FUNYBASE.

#### Finding candidate genes for phylogeny reconstruction

based on the topological scores available in FUNYBASE, one can choose the genes with the appropriate genetic diversity according to the phylogenetic scale sampled (see "Usefulness for phylogenetics").

#### Finding genes with particular evolutionary trends

genes that produce discordant topologies are likely candidates for accelerated evolution or horizontal transfers, which may be associated with important functional divergences. FUNYBASE provides the topological comparison data enabling the detection of such interesting candidate genes.

### Usefulness for phylogenetics

The novelty of FUNYBASE is that it provides a measure for the performance of each gene in estimating the phylogeny of the included species, i.e. the ability of a gene family to yield a robust phylogenetic tree reflecting relatedness defined by larger-scale genomic data and at a variety of taxonomic scales [11]. Several factors may influence this performance, such as the size of the encoded protein, the rate and mode of evolution of the gene and its demographic and selective histories.

We have shown in a previous study that the phylogenetic performance of individual genes is highly variable. Indeed, among the 246 clusters of orthologs, only two gene families yielded, individually, exactly the same topology as the tree based on concatenation of roughly half of the 246 clusters [11]. Interestingly, the genes typically used for fungal phylogenies, encoding gamma and beta tubulins or elongation factors, were not among the best performing genes, as they yielded phylogenies very different from the reference species tree [11]. For studies integrating new fungal samples, genes providing the informational value for phylogenetic reconstruction can be selected [11], economizing on costs of sequencing and improving the accuracy of phylogenies. Genes with high phylogenetic performance will also be of great interest for bar coding (i.e. species identification based on a few DNA sequences).

The phylogenetic performance of the 246 clusters of orthologs was assessed at a large taxonomic scale (Fig. 1), but FUNYBASE can also be used for finding useful genes for building phylogenies at a lower taxonomic scale, such as closely related species or even within species. For this goal, genes with a sufficient degree of divergence at the appropriate taxonomic scale should be chosen, and not necessarily the genes that were found to have the highest phylogenetic performance at the scale of the Fungi. The alignments in FUNYBASE can be used to design primers. We briefly present below two examples of such studies (complete results will be reported elsewhere).

The phylogeny of the genus *Botrytis*, encompassing 22 phytopathogenic species including *B. cinerea*, responsible for the grey mould on many crops, has recently been revised using a phylogeny built based on three nuclear genes [26]. However, several nodes remained poorly supported. In addition, *B. cinerea *was recently shown to be subdivided into two cryptic sympatric species [27], temporarily named *B. cinerea *Group I and Group II, the first being not included in the phylogeny of the genus [26]. We therefore wanted to improve the phylogeny of the *Botrytis *genus, and we searched the complete genome sequences of *Botrytis cinerea *on the websites of URGI  and of the Broad Institute  for genes homologous to FUNYBASE single-copy orthologs that were sufficiently variable for our purpose. Among the 246 single-copy clusters of FUNYBASE, we identified 42 genes from *Botrytis cinerea *(Bc) displaying less than 40% identity at the protein sequence level compared to the corresponding orthologous genes from *Sclerotinia sclerotiorum *(Ss). We designed primers for 3 among the most variable genes: MS401, MS547 and FG1020 (Bc-Ss proteic identities of 23.4%, 25%, and 28.4%, respectively) by aligning nucleotide sequences for each candidate ortholog, extracted from the *B. cinerea *and *S. sclerotiorum *complete genomes, and targeting conserved regions. PCR amplification and sequencing were successful. We sequenced a 808-bp fragment from FG1020 and a 942-bp fragment from MS547 in 23 *Botrytis *species. Both genes exhibit sequence differences among these species, except *B. pelargoni *that was identical to the *B. cinerea *Group II. A well-resolved phylogeny of *Botrytis *species could then be built, with a well-supported placement of the new species *B. cinerea *Group II.

The usefulness of the FUNYBASE database for fungal phylogenetics was also tested using species from *Penicillium *(and *Talaromyces*, the name for the sexual form of *Penicillium*). This group contains mainly soil fungi, and the opportunistic human pathogen, *Penicillium marneffei*. The single previous phylogenetic analysis of this group used the internal transcribed spacers and 5.8S rRNA (ITS1-5.8S-ITS2) sequences [28]. Our aim was to evaluate the extant phylogeny of this group using single-copy sequences and to find genes which could be used for the specific detection of these species which are not always discriminated using their ITS sequences, the common "barcode" in fungi. We used FUNYBASE to retrieve single-copy orthologs with different rates of evolution and we estimated their performance at different taxonomic scales within *Penicillium*. We chose five orthologs with a topological score higher than 91 and with different levels of variability among fungal species: MS277, MS456, MS501, FG610 and FG813. The corresponding protein sequences from *Aspergillus fumigatus*, the closest species to *Penicillium *available in FUNYBASE, were used to retrieve their homologues in the sequences of *Penicillium marneffei *and *Penicillium emmonsii *(= *Talaromyces stipitatus*) available in GenBank. Nucleotide sequences from each candidate ortholog family retrieved in *A. fumigatus, P. marneffei *and *P. emmonsii *were aligned and conserved regions were targeted for designing PCR primers. We successfully amplified and sequenced MS456 and FG610 in all the strains available, while MS501, MS277 and FG813 could be amplified only in some species. Using the sequences obtained, phylogenetic trees were constructed using maximum likelihood for each family of orthologs. MS456, the best gene for recovering a larger-scale phylogeny across fungal groups [11] was not variable enough within the genus *Penicillium*. In contrast, FG610, MS501 and MS277 yielded well-supported trees and should be useful for phylogenetics and bar coding within this genus.

## Conclusion

FUNYBASE constitutes a useful resource for facilitating two types of analyses: (i) comparative studies with reliable clusters of orthologs from a user-defined dataset of fungal genomes, and (ii) phylogenetic reconstruction by choosing the genes with the highest informative value at the desired taxonomic level to be studied in a user-defined fungal group.

## Availability and requirements

The database is available at .

## Abbreviations

AFTOL: Assembling the Fungal Tree of Life; AGD: Ashbya genome database; AIC: Akaike information criterion; BBH: Best bi-directional hit; BIC: Bayesian information criterion; BLASTP: Basic local alignment search tool for proteins; BLASTX: Basic local alignment search tool: search protein database using a protein query; BROAD: Broad Institute; COG: Clusters of orthologous groups; EST: Expressed Sequence Tags; FGI: Fungal Genome Initiative; HMM: Hidden Markov model; InPARANOID: Clusters of Orthologous Groups by the Stockholm Bioinformatics Centre; ITS: Intergenic Transcibed Spacer; JGI: Joint Genome Institute; KOG: Eukaryotic orthologous groups; MCL: Markov Cluster Algorithm; MIPS: Munich information center for protein sequences; MIT: Massachusetts Institute of Technology; NCBI: National Center for Biotechnology Information; NITE: National institute of technology evaluation; PAML: Phylogenetic analysis with maximum likelihood; Perl CGI: Common gateway interface for perl; Perl DBI: Database interface (Standard database interface module for perl); Perl POD: Plain old documentation for perl; RSD: Reciprocal smallest distance algorithm; TIGR: The institute of genomic research; URGI: Genomic-Info research unit; WASABI: Web Accessible Sequence Analysis for Biological Inference; WashU: Washington University.

## Authors' contributions

SM, AGJ and HC were involved in the construction of the protein clusters and in the design and implementation of the database. GA performed the phylogenomics analyses. TG, EF, HC, FR and MHL participated in the design of the study. EF, AG and MLV used the database for building phylogenies. HC, TG, EF, GA and MLV wrote the paper.
